# Accelerating the scoring module of mass spectrometry-based peptide identification using GPUs

**DOI:** 10.1186/1471-2105-15-121

**Published:** 2014-04-28

**Authors:** You Li, Hao Chi, Leihao Xia, Xiaowen Chu

**Affiliations:** 1 Department of Computer Science, Hong Kong Baptist University, Kowloon Tong, Hong Kong; 2 Key Lab of Intelligent Information Processing, Institute of Computing Technology, Chinese Academy of Sciences, Beijing, China; 3 Graduate University of Chinese Academy of Sciences, Beijing, China

## Abstract

**Background:**

Tandem mass spectrometry-based database searching is currently the main method for protein identification in shotgun proteomics. The explosive growth of protein and peptide databases, which is a result of genome translations, enzymatic digestions, and post-translational modifications (PTMs), is making computational efficiency in database searching a serious challenge. Profile analysis shows that most search engines spend 50%-90% of their total time on the scoring module, and that the spectrum dot product (SDP) based scoring module is the most widely used. As a general purpose and high performance parallel hardware, graphics processing units (GPUs) are promising platforms for speeding up database searches in the protein identification process.

**Results:**

We designed and implemented a parallel SDP-based scoring module on GPUs that exploits the efficient use of GPU registers, constant memory and shared memory. Compared with the CPU-based version, we achieved a 30 to 60 times speedup using a single GPU. We also implemented our algorithm on a GPU cluster and achieved an approximately favorable speedup.

**Conclusions:**

Our GPU-based SDP algorithm can significantly improve the speed of the scoring module in mass spectrometry-based protein identification. The algorithm can be easily implemented in many database search engines such as X!Tandem, SEQUEST, and pFind. A software tool implementing this algorithm is available at http://www.comp.hkbu.edu.hk/~youli/ProteinByGPU.html

## Background

High-throughput tandem mass spectrometry (referred to hereafter as MS/MS) based protein identification is a powerful method in proteomics [1]. It enables large-scale analysis of the protein sequence and PTMs with high sensitivity, accuracy, and throughput [2-6]. Among the MS/MS data analysis methods, protein database search approaches, such as Mascot [7], SEQUEST [8], pFind [9-11], X!Tandem [12], OMSSA [13], and Phenyx [14], have been the most widely used. Although much research has been devoted to improving the method’s effectiveness by designing new scoring and validating algorithms, creating efficient database search engines is a serious challenge for a number of reasons.

First, the number of entries in a protein sequence database increases continuously. Take, for example, IPI.Human, in which the number of proteins increased by almost a third between v3.22 and v3.49 [15]. Second, if semi- or non-specific digestion is considered, as it increasingly is, it will lead to 10 or 100 times more digested peptides, respectively, in the database, than if only specific digestion is considered. Third, post-translational modifications (PTMs) generate exponentially more modified peptides. Until recently, over 900 types of PTMs existed in the Unimod protein modification for mass spectrometry [1]. If we choose ten common variable PTMs and limit the number of modification sites in a peptide to no more than five, the number of tryptic peptides of the human proteome will be increased over 1000 times. At the same time, the generation speed of the mass spectrometers has been steadily increasing.

As all of the algorithms in a database search engine calculate the similarity between the experimental MS/MS and the theoretical candidate MS/MS generated from the protein (peptide) database, one of the direct results of the above increases is the large scale of the scoring calculation, which is the most computing intensive and time consuming stage in the whole flow of protein identification. Profiling analysis shows that the scoring module takes around 50-90% of the total identification time in both pFind and X!Tandem. Thus, speeding up the scoring module is a promising method to increase the efficiency of protein identification.

Recent studies on efficiency focus on decreasing the redundant scoring operations and parallelizing the scoring module. Some studies adopted indexing techniques to avoid unnecessary scoring. Li and Chi systematically explored the effect of indexing techniques and designed an inverted index strategy for protein identification [15]. Edwards and Lippert considered the problem of redundant peptides and peptide-spectrum matching and used a suffix tree index [16]. Tang adopted peptide and b/y ions indices [17]. Dutta and Chen used the nearest neighbor search to improve peptide-spectrum matching [18]. At the same time, most of the popular peptide and protein search engines use parallel computing technology. SEQUEST adopted a parallel virtual machine (PVM) to build its cluster system [19], whereas Mascot and Phenyx used a message passing interface (MPI). X!Tandem has two parallel strategies [20,21]. These systems have been integrated into higher-level application frameworks, such as web services, grids [22], or even cloud computing. Halligan has migrated X!Tandem and OMSSA to the Amazon cloud computing platform [23]. In addition, some recent systems used new hardware to increase the parallelizing of the scoring module. Bogdan and Dandass use a field-programmable gate array (FPGA) [24]. Hussong used a single graphics processing unit (GPU) to speed up the feature selection step [25]. Baumgardner implemented a spectrum library search algorithm on a single GPU [26]. Milloy also adopted a single GPU to speedup database spectral matching [27].

Recently, GPUs have become general purpose and high performance parallel hardware and provided another promising platform for parallelizing the scoring function. GPUs are dedicated hardware for manipulating computer graphics. Due to the large demand for real-time computing and high-definition 3D graphics, GPUs have evolved into highly parallel many-core processors [28]. NVIDIA GTX580 is an example of a typical GPU architecture. GTX580 has 16 streaming multiprocessors (SMs), and each SM has 32 scalar processors (SPs), resulting in a total of 512 processor cores. The SMs have a single-instruction multiple-thread (SIMT) mode; at any given clock cycle, each SP executes the same instruction, but operates on different data. The recent advances in computing power in GPUs have driven the development of general-purpose computing on GPUs (GPGPU), which have been used to accelerate a wide range of applications [29-33].

Considering the independence of each scoring operation in a protein identification database search engine, it is reasonable to parallelize the scoring module in an SIMT architecture on a GPU or GPU cluster. To the best of our knowledge, three studies [25-27], have attempted to use GPUs to speed up peptide/protein identification. Hussong et al. [25] focused on peak selection, while [26] and [27] are dedicated on spectral library search. Meanwhile, few studies have discussed peptide/protein identification on a GPU cluster. For this study, we choose one of the most widely used scoring methods, spectral dot product (SDP), which can be used directly or indirectly in X!Tandem, pFind, SEQUEST, etc. We conduct systematic research to design a parallel SDP-based scoring module for both a single GPU and a GPU cluster, using a general purpose parallel programming model, specifically, the Compute Unified Device Architecture (CUDA).

Our first contribution is the design, implementation, and evaluation of two different parallel SDP algorithms on a GPU, based on the precursor mass distribution of the experimental spectrum. The precursor mass distribution describes the number of spectra in a group of preset consecutive mass windows, and marks the windows as sparse or dense. For the sparse mass windows, we use the GPU on-chip registers to minimize the memory access latency. However, due to the limited size of the on-chip registers, this method is not applicable to the dense mass windows. Consequently, we design a novel and highly efficient algorithm that treats the experimental and theoretical spectra as two matrices, and considers the scoring process as a matrix operation, and then makes use of the GPU on-chip shared memory together with the on-chip registers. Using both of the above two algorithms, we achieve a 30 to 60 times speedup compared to the serial version on a single CPU.

Our second contribution is the adoption of a GPU cluster for protein identification that uses a novel pre-calculation strategy to balance the workload on each node and to decrease the communication costs between the master node and worker nodes. We consider the operation number of each scoring process between the theoretical and experimental spectra as the basic task, divide the mass range into sub-mass ranges where the number of the basic operation in each sub-range is almost the same, and then dispatch the task to the sub-range. In the end, we obtain a favorable speedup on our GPU cluster that contains eight Nvidia GTX580 GPUs with a total of 4096 processing cores.

## Results

**Figure 1:**
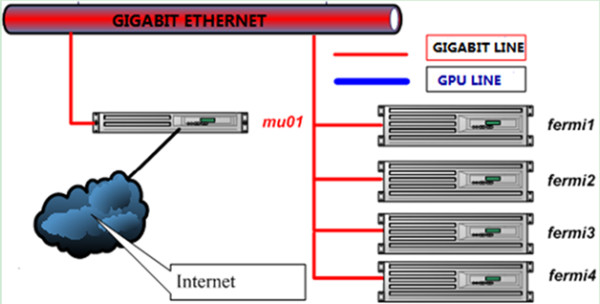
**GPU cluster architecture.** A GPU cluster has one master (mu01) and four computing nodes (Fermi.1-4). All of the nodes have a XeonE5620, and perform at 2.40 GHZ with two GeForce GTX580. The GTX580 has 512 cores, performs at 1.54 GHz, and has 1.54GB global memory with a peak bandwidth of 192.4 GB/sec. All of the nodes are connected by the GIGABIT line, and mu01 is connected to the Internet.

All the experiments were conducted on a GPU cluster that included one master node (mu01) and four computing nodes (Fermi.1-4), as shown in Figure 1 and Table 1. All of the nodes had a Xeon E5620 CPU performing at 2.4 GHz, and two NVidia GeForce GTX580 cards. Each GTX580 had 512 cores, performing at 1.54 GHz and with a peak memory bandwidth of 192.4 GB/sec. The CPU-based programs were developed by C++ language, and the GPU-based program used CUDA 4.2.

**Table 1:** GPU cluster specifications

**Node**	**Mu01**	**Fermi1-4**
GPU	N/A	2 × GTX580
CPU	2 × XeonE5620(2.40GHz)/5.86GT/12 M/1066	
Memory	6 × 4G Registered ECC 1333 MHz DDR3	
Others	1 × 1000G 3.5inch SATA, 2 × 1000 M Ethernet	

We performed three experiments to show the speedup effect, using the searching parameters in Table 2. The MS/MS data in Exp.1 were downloaded from a previously reported dataset [34], generated from QSTAR instrument. This dataset was used to evaluate the performance of the target-decoy approach, which was one of the most classic and important works for the evaluation of peptide identification results. In Exp.2, the MS/MS data were generated by another liquid chromatography/tandem mass spectrometry (LC/MS/MS) experiment that analyzed a mixture of human serum proteins. In Exp.3, the MS/MS data and searching parameters were the same as Exp.1, but were searched against UniProtKB/Swiss-Prot (2013.05.15) database.

**Table 2:** Database searching parameters

Exp. 1	Instrument	QSTAR
	Spectra	46195 DTA files
	Database	Yeast (13434 proteins, target + reversed)
	Enzyme	Trypsin (max missed cleavage sites = 2)
	Tolerance	Precursor: 0.2 Da; Fragment: 0.2 Da
	Modifications	Fixed: Carbamidomethylation (C)
		Variable: Oxidation (M), Phosphorylation (S, T, Y)
Exp. 2	Instrument	LTQ
	Spectra	43493 DTA files
	Database	IPI.Human v3.49 ( 148034 protein, target + reversed)
	Enzyme	Trypsin (max missed cleavage sites = 2)
	Tolerance	Precursor: 3 Da; Fragment: 0.5 Da
	Modifications	Fixed: Carbamidomethylation (C)
		Variable: Oxidation (M), Phosphorylation (S, T, Y)
Exp.3	Instrument	QSTAR
	Spectra	46195 DTA files
	Database	UniprotKB/Swiss-Prot (540171 proteins)
	Enzyme	Trypsin (max missed cleavage sites = 2)
	Tolerance	Precursor: 0.2 Da; Fragment: 0.2 Da
	Modifications	Fixed: Carbamidomethylation (C)
		Variable: Oxidation (M), Phosphorylation (S, T, Y)

We mainly considered the scale of the spectra and protein database to test the speed, while Exp.1, Exp.2 and Exp.3 could be considered as small, large and medium computing scale respectively. We also considered the mass distribution of the matched spectrum-peptide, which was a concern when we designed the speedup algorithm, and analyzed in the next section: *SDP on a single GPU*.

### SDP on a single GPU

We first performed the experiment on X!Tandem (win.2011.12.01) and pFind (V2.6.0) using the Fermi.1 (Xeon E5620 CPU). Both X!Tandem and pFind versions were serial and single thread program adopting CPU only. The running time results were shown in Table 3. In Exp.1, X!Tandem spent 45 minutes in total, of which 26 minutes were for the scoring function, namely “*dot*()” in the source code, which computed the SDP and occupied 58% of the total time. Similarly, pFind used 18 out of 22 minutes, which was 82% of the total time, on the scoring function “*ksdp*()”. The time distribution in Exp.2 shared the same characteristics, and demonstrated the potential for increasing efficiency by parallelizing the scoring module. It is also worth pointing out that many optimization methods can be used to speed up the modules other than the scoring module [14,16-18]. Our work is complementary to those methods.

**Table 3:** Time usage of database searching (minutes)

**Search engines**	**Time distribution**	**Exp. 1**	**Exp. 2**	**Exp. 3**
X!Tandem	Total time	45	1011	253
	Scoring time	24	566	138
	Scoring time percentage	54%	56%	55%
pFind	Total time	22	601	132
	Scoring time	18	530	107
	Scoring time percentage	82%	89%	81%

Note: pFind and X!Tandem both use a one-step mode.

We implemented single thread/process CPU SDP version Algorithm 1, and serially executed on the Fermi.1. We also implemented single GPU SDP version Algorithm 2 and 3, and executed on the Fermi.1. Ignoring the time of reading database and spectra files for all the algorithms, the speedup from a single GPU (Fermi.1) varied from thirty to sixty-five, as shown in Table 4. Exp.1 achieved a 31 times speedup, Exp.2 achieved a 65 times speedup, Exp.3 achieved a 29 times speedup. The speedup effect resulted from the parallel scoring and memory access optimization.

**Table 4:** Speedup effect of SDP using a single GPU

**Search engines**	**Exp. 1**	**Exp. 2**	**Exp.3**
CPU	968 s	32587 s	5529 s
GPU	35 s	502 s	191 s
Speedup	27	65	29

Note: in Exp.1, *threshold* is set to 1, whereas in Exp. 2, *threshold* is set to 2.

We implemented these two algorithms on the GPU with the following strategy. First, we calculated the precursor mass distribution of the experimental spectra, and counted the spectra number in a consecutive group of 1 Da mass windows from 300 Da to 4000 Da, like 300 ~ 301 Da, 301 ~ 302 Da, …, and 3999 ~ 4000 Da. Second, we divided the mass window into two categories: if the number of experimental spectra was not larger than a preset *threshold* number in a mass window, then it was a *sparse window*; otherwise it was a *dense window*. Take Exp.1 as an example. The *threshold* was set to two and 8.3% of the mass windows are sparse. For dense windows, the average number of experimental spectra was twenty-one. Third, we adopted Algorithm 2 to handle sparse windows and used Algorithm 3 to handle dense windows.

Algorithm 2 exploited the on-chip registers, to decrease the memory access latency. On GTX580, each SM has 32,768 registers, and registers have 32 bits. Each theoretical spectrum need around 16 registers on average, in Exp.1. We can infer that each SM could store around 2,048 spectra, and 16 SM could deal with 32,768 experimental spectra on the register. In addition, Algorithm 2 stored the experimental spectra on the texture memory, which used a cache mechanism to decrease the memory access latency. Algorithm 2 also put the index file for the theoretical and experimental spectra mating on the constant memory to further decrease the reading latency. Consequently, Algorithm 2 read the theoretical spectra from global memory only once; then it read experimental spectra from global memory also once, and read from texture less than *threshold* times from global memory, which was two in Exp.1 and one in Exp.2; then it loaded theoretical spectra into the register, and calculated the score of these experimental spectra. We presented the idea in detail in Algorithm 2.

For the experimental spectrum in the dense mass window, Algorithm 2 will not work because there are not enough registers. Instead, we designed Algorithm 3 to adopt a shared memory that is larger than the registers; the reading latency is also much better than reading from the global memory. Algorithm 3 considered the spectrum in the dense mass window as a matrix, and loaded the theoretical spectrum matrix, tile by tile, into the shared memory. Thus it accessed the global memory only once for each theoretical spectrum. Consequently, on average, Algorithm 3 read both the experimental and theoretical spectra from global memory once. If we still use the Algorithm 2 here, we would read the theoretical spectrum from local memory, for 21 times in Exp.1. on average. Besides, Algorithm 3 also used the constant memory for the index file.

To illustrate the utility of our mixed design strategy, we also compared the speedup effect of adopting Algorithm 2 or Algorithm 3 alone. In Exp.2, Algorithm 2 spent 8,427 seconds while Algorithm 3 spent 936 seconds. Both were not as efficient as the mixed strategy. Besides, Algorithm 2 performed much worse when it had to reading from the local and global memory multiple times, since the reading latency of local and global memory is much longer than that of the register. On the other hand, Algorithm 3 mainly made use of the shared memory, whose reading latency is small than local and global memory, and a little longer than the register when we avoided the banking conflict. In the Methods section, we showed how Algorithm 3 made the most use of the shared memory.

### SDP on the GPU cluster

We designed two parallel SDP algorithms: one adopted CPUs alone, namely CPU Cluster version, while the other one adopted both CPUs and GPUs, namely GPU cluster version. We divided the parallel SDP algorithm into two steps: in the step 1, we assigned a sub task to one computing node and prepared the database and spectra in each node; in the step 2, we calculated the SDP in each node on its own task. We designed a pre-calculation strategy for the task assignment, adopted algorithm 1 for calculating the SDP on the CPU cluster, and used Algorithm 2 and 3 for calculating the SDP on the GPU cluster.

In the experiment, we copied all the databases and spectra in each node (Fermi1-4) first, calculated the sub task on mu01, sent messages (MPI) to each node, and calculated the SDP. As shown in Table 5, the speedup of the GPU cluster version, compared with CPU cluster version, varied from thirty to seventy times. The speedup came from both of the two steps. In the step 1 for pre-calculation, we got eight times speedup in Exp.1, and thirty times in Exp.2. In the step 2 for SDP calculation, we got 35 times speedup in Exp.1, and 71 times in Exp.2, resulting from the same reason in the previous section, SDP on the single GPU. The time consumption of step 1 was less than 10% in the CPU cluster versions, and the direct reason of the above speedup came from the second step. On the other hand, the strategy in step 1 created a promising overflow balance and achieved a favorable speedup in both the CPU- and GPU-cluster versions, compared to the single node version, as shown in Table 6.

**Table 5:** Speedup effect of SDP using the GPU cluster

**Search engines**	**Exp. 1**		**Exp. 2**		**Exp. 3**	
	**Scoring**	**Pre-calculation**	**Scoring**	**Pre-calculation**	**Scoring**	**Pre-calculation**
CPU-cluster	273 s	14 s	8991 s	242 s	1568s	94 s
GPU-cluster	13 s	3 s	136 s	8 s	62 s	5 s
speedup	21	5	66	30	25	19

Note: we use one CPU and one GPU in each node of the cluster.

**Table 6:** Speedup effect of the pre-calculation strategy in Exp.2

**Node number**	**CPU-cluster**			**GPU-cluster**		
	**Scoring**	**Pre-calculation**	**Speedup percentage**	**Scoring**	**Pre-calculation**	**Speedup percentage**
one	32587 s	242 s		502 s	8 s	
two	17997 s		89.3%	279 s		87.4%
three	12153 s		87.6%	178 s		89.9%
four	8991 s		88.2%	136 s		87.1%

Note: speedup percentage equals to: one scoring time/ (N node scoring time + pre calculation time)/N.

**Figure 2:**
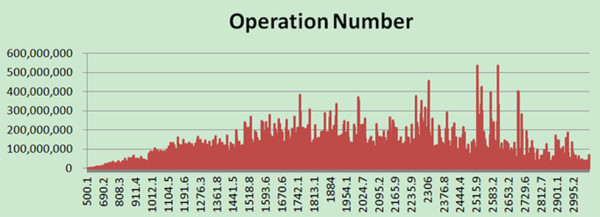
**The number of operations in each 0.1 Da mass window, from 300 Da-4000 Da, in Exp. 1.** The x-axis stands for the mass range; divide the mass range, from 300 Da to 4000 Da, into 36000 equal-sized 0.1 Da mass windows. The y-axis stands for the operation number between the experimental and theoretical spectrum in each mass window.

The pre-calculation strategy first calculated the operation numbers of each scoring between the experimental- and theoretical- spectrum in the mass window, where any addition or multiplication was considered to be one operation. The result of Exp.1 was shown in Figure 2. The Methods section presented the detailed calculation algorithm. Based on this operation distribution, the work can be equally divided into *N*_*G*_ mass ranges, where *N*_*G*_ stood for the number of GPUs in our cluster. In each mass range, each GPU got nearly the same work, and this ensured a good workload balance. The cost of our strategy was the calculation of operation number, which was nearly the same as the workflow of protein identification before the scoring stage. The time consumption is around 4% of the scoring time in the CPU version, and 6 ~ 10% in the GPU version. Obviously, the more GPU nodes we adopt, the lower cost this strategy achieves.

Normally, the strategy for the cluster is based on the spectrum. The master node sends a preset number of spectra to each worker; if one worker has finished its spectra, then the worker asks for the next group from the master node. However, the amount of work on each worker node might be significantly different. In Exp.1, the experimental spectrum in the precursor mass window with 1105.5 ~ 1105.6 Da, scored with 3157 theoretical spectra, whereas the experimental spectrum with precursor mass 522.3 ~ 522.4 Da scored with 23 theoretical spectra. Another more careful strategy is based on the scoring number of each spectrum; each worker deals with the same number of the scoring process. However, different scoring process could have very different operations. For example, assuming peaks are fully matched, the SDP operation number is 20 for a matched spectrum pair with 5 hit peaks, whereas the number is 200 for a matched spectrum pair with 50 hit peaks. As a result, the above methods do not balance the workload on each worker very well. The communication between the master and the worker is also higher than in our strategy.

## Results and discussion

GPU-SDP does not compromise on the accuracy, and can be easily integrated into many search engines. Besides, it can also be very easily enhanced to support other similar scoring methods, such as XCorr, KSDP, or other probability-based methods. In the future, we will implement a complete GPU cluster-based search engine for protein identification, and the estimated initial effect could be seen in Additional file 1.

## Conclusions

In this study, we present a novel GPU-based scoring method, and design and implement a SDP-based scoring module on a GPU platform. We achieve an approximate 30 to 60 times speedup on a single GPU node, relative to a serial CPU version, and a favorable speedup effect with a GPU cluster with four nodes.

## Methods

The basic notations are as follows: *T* and *C* are the theoretical spectra set and the experimental spectra set; *T*_*i*_ and *C*_*i*_ are the *i*-th element in *T* and *C*, stores the *m/z* values, and are described as vector *T*_*i*_ = [*t*_*i*_*1*_, *t*_*i_2*_,…, *t*_*i_Nt*_] and *C*_*i*_ = [*c*_*i_1*_, *c*_*i_2*_, …, *c*_*i_Nc*_], where *N*_*t*_ and *N*_*c*_ are the number of different *m/z* values; and *t*_*i_j*_ and *c*_*i_j*_ are the *j*-th *m/z* value in the MS/MS spectrum; *T’*_*i*_ and *C’*_*i*_ are also the *i*-th element in *T* and *C*, stores the *intensity* values, and are described as vector *T’*_*i*_ = [*t’*_*i_1*_, *t’*_*i_2*_, …, *t’*_*i_Nt*_] and *C’*_*i*_ = [*c’*_*i_1*_, *c’*_*i_2*_, …, *c’*_*i_Nc*_], where *t’*_*i_j*_ and *c’*_*i_j*_ are the *j*-th *intensity* value in the MS/MS spectrum.

The workflow of the scoring module contains three steps, as shown in Algorithm 1. First, line 1 and 2 perform the theoretical and experimental spectrum matching. For each theoretical spectrum *T*_*i*_, the algorithm will search all of the experimental spectra whose precursor masses are in the peptide’s precursor mass window and will get C^*^. We adopt the spectrum hash indexing technology presented in our previous study [15] to find the matched spectrum in O(1) complexity, where the cost of the indexing is O(|*C*|).

Second, lines 4–7 conduct the peak matching of each matched theoretical and experimental spectrum pair. For each peak in the theoretical spectrum, the algorithm will search for the first matched peak in the experimental spectrum and get *T’*_*i*_ = [*t’*_*i_1*_, *t’*_*i_2*_, …, *t’*_*i_N*_] and *C’*_*i*_ = [*c’*_*i_1*_, *c’*_*i_2*_, …, *c’*_*i_N*_], where *N* is the number of matched peaks, and *t’*_*i_j*_ and *c’*_*i_j*_ are the intensity of the *j*-th matched peak (*t’*_*i_j*_ and *c’*_*i_j*_ could also be valued as 1). We again adopt the spectrum peak hash indexing technology from our previous study [15] to find the matched peak in O(1) complexity. The cost of the indexing is O(*N*_c_), and the complexity of the peak matching is O(*N*_c_ *+ N*_t_).

Third, lines 6, inside the second step, computes the *SDP* value by the matched peaks for each matched theoretical and experimental spectrum pair. The *SDP* value is defined as Equation (1), where *N* is the number of hit peaks. Based on the above three steps, the whole computation complexity is O(|*C*| + |*T*||*C’*|( *N*_c_ + *N*_t_)).

(1) SDP=<T'i,C’j>=∑i=1Nt'ic'i

### SDP on the single GPU

In the CUDA model, the GPU is considered a coprocessor that is capable of executing a large number of threads in parallel. The GPU threads are organized into thread blocks, and each block of threads is executed concurrently on one streaming multiprocessor (SM). Each SM has four different types of on-chip memory, namely registers, shared memory, constant cache, and texture cache [31]. Constant cache and texture cache are both read-only memories shared by all of the scalar processors (SPs). Off-chip memories, such as local memory and global memory, have more space but relatively long access latency, usually 400 to 600 clock cycles [35]. The properties of the different types of memory are summarized in [35,36]. In general, the scarce registers and shared memory should be carefully used to amortize the global memory latency cost.

Our first SDP algorithm on a GPU is written so that each thread deals with one theoretical spectrum, scoring with its entire matched experimental spectrum, as shown is Algorithm 2. The differences between Algorithms 1 and 2 are as follows. First, Algorithm 2 unfolds the first *for* in Algorithm 1, by assigning each theoretical spectrum to a thread, which decreases the time consumption significantly as many threads are working in parallel. Second, Algorithm 2 merges the peak matching and SDP calculation steps to decrease the space for the variable.

When implementing Algorithm 2, we first copy the theoretical spectrum to the global memory, then store the experimental spectrum on the texture memory and put the spectrum index file on the constant memory. We notice that when the spectrum dataset is small, including the total number and the spectrum length, we can use the on-chip register for the experimental spectrum and other variables. As Algorithm 2 reads a theoretical spectrum | C^*^| times, where | C^*^| stands for the number of theoretical spectra scoring the experimental spectrum, reading from the register can significantly reduce the reading latency. We illustrate the effect in detail in the *Results* section.

However, the problem in the implementation of Algorithm 2 is the limited size of the registers. In fact, users are not allowed to fully control the registers, and can only adopt registers when the data size is small enough. As the size and length of the spectrum grows, the data cannot be loaded into the registers and are instead stored in local memory or global memory, which increases the reading latency and decreases the performance significantly.

In each mass tolerance window, a group of experimental spectra will score with a group of theoretical spectra. Take Exp.1 as an example. On average, 14,880 theoretical spectra will score with 21 experimental spectra in a one Dalton mass window with a range of 300 ~ 4000 Da. Thus, the theoretical and experimental spectra could be considered two matrixes, *theo*[|T^*^|][*N*_*t*_] and *expe*[*N*_*c*_][|C^*^|], the result score could be denoted as *Scor*[|T^*^|][|C^*^|], and the score calculation process could share a similar flow as the matrix multiplication. Based on this observation, we design Algorithm 3 for a dense mass, using registers and shared memory together.

**Figure 3:**
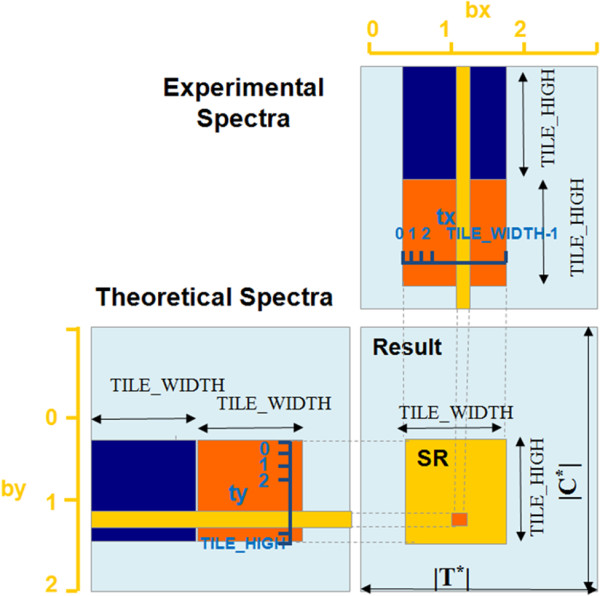
**The computing process in a dense mass window.** The figure shows the calculation in one dense mass window. The result is a *Scor*[*TH*][*TW* × *TDimY*], which is equal to *theo*[*TH*][*N*_*t*_] × *expe*[*N*_*c*_][*TW* × *TDimY*]. Load the first tile (in blue) from the *theo* into the shared memory; score the blue tile in the *theo* with the blue tile in the *expe*, which is stored in the texture memory; accumulate the temporary results into *TResult*, whose initial value is zero; then repeat loading the next tile (in orange), scoring and accumulating, until *theo*[*TH*][*N*_*t*_] and *expe*[*N*_*c*_][*TW* × *TDimY*] have all been accessed.

As shown in Algorithm 3, each of the mass window matrixes *theo*[|T^*^|][*N*_*t*_], *expe*[*N*_*c*_][|C^*^|], and *Scor*[|T^*^|][|C^*^|] are partitioned into *TH × TW*, *TW × TH*, and *TH × TW* tiles, respectively, where *N*_*t*_ and *N*_*c*_ are the maximal length of the experimental and theoretical spectra. *TH* and *TW* are preset values, which could be the integral multiple number of the thread number in a half GPU warp, 16, 32 or 64, to make the best use of the GPU warp mechanism. The resources of the GPU are partitioned as follows: the grid has (|C^*^|*/TW*) *×* (|T^*^|*/TH*) blocks, the ID of which is noted by *blockIdx.y* (*by* in Figure 3) and *blockIdx.x* (*bx* in Figure 3); and each block has *TH* × *TDimY* threads, the ID of which is noted by *threadIdx.y* (*ty* in Figure 3) and *threadIdx.x* (*tx* in Figure 3). The computing task is dispatched as follows: each block calculates *TDimY* tiles in the *Scor*, which is noted as *SR*[*TH*][*TW* × *TDimY*]; then each thread computes a column of *SR*. For each thread, *indexT* points to the right position of the theoretical spectrum, which contains the following three parts as shown in line 4: *theo* is the beginning address of the theoretical spectrum; as the height of the *theo* is divided by *TH*, *blockIdx.y* × *TH* × *N*_*t*_ is the address of the corresponding block; and *threadIdx.y* × *N*_*t*_ adding *threadIdx.x* is the offset address inside the block.

In line 5, *indexC* points to the right position in the experimental spectrum, which also has three parts: *expe* is the beginning address of the current spectrum; *blockIdx.x* × *TW* points to the corresponding block address, as the width of the experimental spectrum is divided by *TW*; and *threadIdx.y* × *blockDim.x* adding *threadIdx.x* points to the address of the current thread inside the block. Obviously, the threads in one block would access the experimental spectrum in continuous addresses, which is also called coalesced accessing. *indexR* is calculated in the same way as in line 6 using the beginning address of the result, the row address, and the offset address inside the block for the current thread.

In the loop from line 11 to 16, the algorithm loads a tile of data from the global memory to the shared memory, and computes the SDP score saved in *TResult,* which is stored on the on-chip registers; the loop ends when the whole row has been calculated. Line 17 waits for all of the threads to finish their work. Line 18 writes the distance back from *TResult* to *SR.* The details are shown in Figure 3, which takes the process of calculating a *Scor*[*TH*][*TW* × *TDimY*] as an example. It is equal to *theo*[*TH*][*N*_*t*_] × *expe*[*N*_*c*_][*TW* × *TDimY*] and the sequence is the following. Load the first tile (in blue) from the *theo* into the shared memory; score the blue tile in the *theo* with the blue tile in the *expe*, which is stored in the texture memory; accumulate the temporary results in *TResult*, whose initial value is zero; then load the next tile (in orange), and continue scoring and accumulating until *theo*[*TH*][*N*_*t*_] and *expe*[*N*_*c*_][*TW* × *TDimY*] have been all accessed.

The main purpose of Algorithm 3 is to decrease the global memory access time and latency by loading the theoretical spectrum into the shared memory, tile by tile. Thus, Algorithm 3 reads each theoretical spectrum from global memory only once, the same as Algorithm 2. The key feature of Algorithm 3 is how efficiently it accesses the global memory and shared memory; this is achieved by adopting coalescing reading that accesses sixteen continuous addresses for the threads in a half warp to avoid the bank conflict.

### SDP on the GPU cluster

On the GPU cluster, as each node could adopt Algorithm 2 and 3, the main concern is how to dispatch the work to each node and achieve a high workload balance. We design a new complete pre-calculation strategy to make each node work on nearly the same task. In this strategy, we first run the workflow of protein identification before the scoring stage to get experimental and theoretical matching results. These results tell us how many peptides each spectrum will score with, as shown in Algorithm 4, line 1–4.

Second, in line 5 of Algorithm 4, we calculate the operation number for each SDP scoring in the following way: *d*_*e*_ + *d*_*t*_ + *hitcount* × 2, where *d*_*e*_ and *d*_*t*_ are the dimension of the experimental and theoretical spectra, and *hitcount* is the number of hit peaks. *d*_*e*_ + *d*_*t*_ stands for the peak matching step in Algorithm 2, and *hitcount* × 2 stands for the dot product step in Algorithm 2. As a result, we get the operation number for each mass range, such as one Dalton, from 300 Dalton to 4000 Dalton, based on the experimental and theoretical precursor mass, matching results, and each SDP operation.

Third, we dispatch the task by mass range and give each node the same amount of work. As shown in Algorithm 5, line 1–3 calculate the total number of operations; line 4 gets the average number of operations on each GPU node; line 5–10 travers the *Oper* array; when the temporal summary of operation exceeds the average number *WorkerOper*, Algorithm 5 call Algorithm 2 or 3, to deal with the current mass range, which is *Oper*[*p*] to *Oper*[*k*].

The overhead of the pre-calculation strategy is also very low, as shown in the Results section. After the calculation, the master node only transfers data to the computing node once, and this lowers the communication cost.

## Competing interests

In the past five years, all the authors have not received reimbursements, fees, funding, or salary from an organization that may in any way gain or lose financially from the publication of this manuscript. And all the authors will not receive them in the future. No such organization is financing this manuscript (including the article-processing charge.

All the authors do not hold any stocks or shares in an organization that may in any way gain or lose financially from the publication of this manuscript, both now and in the future.

All the authors do not hold and are not applying for any patents relating to the content of the manuscript.

All the authors have not received reimbursements, fees, funding, or salary from an organization that holds or has applied for patents relating to the content of the manuscript.

All the authors do not have any other financial competing interests.

There are not non-financial competing interests (political, personal, religious, ideological, academic, intellectual, commercial or any other) to declare in relation to this manuscript.

## Authors’ contributions

YL and XC designed this study. YL implemented the algorithms and performed the experiment. YL, HC, and LX implemented the algorithms in the software. YL, HC, and LX analyzed the data. All of the authors have read and approved the final manuscript.

## Supplementary Material

Additional file 1 Initial accelerating effect of the peptide identification search engine using GPUs. Click here for file
