# Supplementary material for: Accelerating the scoring module of mass spectrometry-based peptide identification using GPUs
Source: BMC Bioinformatics. 2014 Apr 28;15:121. doi: 10.1186/1471-2105-15-121 (PMC4049470; doi:10.1186/1471-2105-15-121)
Supplement: Additional file 1 — Initial accelerating effect of the peptide identification search engine using GPUs. [file 1471-2105-15-121-S1.pdf]

In this supplementary, we analyze the speedup effect on the entire peptide identification search engine. As shown in STable 1, we divided the whole peptide identification process into four modules, namely scoring, dispatching, validation, and others [32]. ***Dispatching*** module stands for the generation of (modified) peptides and the matching between the peptides and the spectrum; ***Scoring*** module refers to the calculation of SDP between the matched peptides and spectrum; ***Validation*** module evaluates the identified results; ***Others*** module includes the spectrum pre-processing, input, and output. In STable 2, we could see that the overall speedup achieved by our GPU-based SDP ranges between 2.3x and 4.4x.

STable 1 time distribution of pFind

Exp./percentage	Total time	Scoring	Dispatching	Validation	Others
Exp.1	22 m	18m, 82%	8%	3%	7%
Exp.2	601 m	530m, 89%	5%	2%	4%
Exp.3	132 m	107m, 81%	10%	3%	6%

STable 2 time distribution of pFind

Exp./percentage	Scoring time On CPU	Scoring time On GPU	Total time on CPU	Total time on GPU	Speedup effect
Exp.1	18m	0.52m	22 m	5 m	4.4
Exp.2	530m	82m	601 m	153 m	3.9
Exp.3	107m	32m	132 m	57 m	2.3

Compared with the significant speedup effect of the scoring module only, we could not get the similar favorable result on the whole search engine due to the limitation of Amdahl's law. In order to further improve the overall speedup, we plan to accelerate other modules such as dispatching, spectrum pre-processing, and protein database indexing in our future work.
